# Drug-resistant *Plasmodium falciparum*: are recent advances a cause for optimism?

**DOI:** 10.2217/FMB.15.58

**Published:** 2015-07-30

**Authors:** Timothy J Egan

**Keywords:** artemisinin-resistance, chloroquine, chloroquine-resistance, dihydroartemisinin, K13-propeller domain, malaria, PfCRT, phosphatidylinositol-3-kinase, phosphatidylinositol-3-phosphate

The period since 2000 has seen dramatic advances in the fight against malaria. The disease has been eliminated in 11 countries and is in the elimination or pre-elimination phase in 19 others. In addition, by 2013 there had been a 26% decrease in malaria cases, a 46% decrease in prevalence of childhood malaria and a 47% decrease in malaria deaths worldwide since 2000. These advances can be attributed to several strategies, notably the use of insecticide treated bed nets, indoor residual spraying of insecticides, intermittent preventative treatment and improved diagnosis and treatment of malarial infections. In the case of treatment, the use of artemisinin combination therapy has been shown to reduce childhood mortality by an average of 97 to 99%. This remarkable progress can be attributed to the commitment of resources. In the period 2005–2013 funding for malaria control and elimination increased almost threefold, with the three largest sources being the Global Fund to Fight AIDS, TB and Malaria, the United States President's Malaria Initiative together with USAID and the governments of malaria endemic countries themselves [1]. The tools that have been developed to control and eliminate malaria and the funds devoted to this cause have in many ways rivaled the achievements of the 1950s and 60s, which were brought about by the use of DDT and chloroquine (CQ). Ominously, however, in much the same way as CQ-resistance began in southeast Asia near the Thai–Cambodia border approximately 55 years ago, so artemisinin-resistance has become apparent in the same region in the last 5 years [2,3]. This was later shown to have spread to the Thai–Myanmar border area [4]. While artemisinin combination therapy largely remains effective, evidence soon emerged of increased failure rates of artesunate mefloquine combination therapy linked to artemisinin-resistance near the Thailand–Cambodia border [5]. Thus the problem of artemisinin resistance represents a serious risk to the future success of the malaria control and elimination program.

It is now widely accepted that mutations in the gene encoding PfCRT, a digestive vacuole (DV) transporter located in the DV membrane is the primary factor in CQ resistance in *Plasmodium falciparum*, albeit that other proteins probably also contribute to the level of resistance [6]. Discovery of the *pfcrt* gene and its role in CQ resistance only occurred 40 years after the first appearance of resistance [7], by which time it was almost universal. Even today, the natural physiological role of PfCRT is not known. In the case of artemisinin, clinical evidence for resistance was first published in 2008 [2]. Molecular markers for resistance in the Cambodian strains were identified within 6 years [8]. Using whole-genome sequencing of an artemisinin-sensitive parasite line from Tanzania cultured under escalating drug pressure for 5 years and clinical artemisinin-resistant isolates from Cambodia, Ariey *et al.* were able to identify mutations in the PF3D7_1343700 kelch propeller domain, or PfKelch13, as markers of artemisinin-resistance. Three PfKelch13 polymorphisms (C580Y, R539T and Y439H) were found to be correlated with significant increases in parasite clearance time in patients, with the C580Y mutation showing the largest increase. These changes, especially the C580Y polymorphism, were found at high frequency in provinces of western Cambodia where clinical artemisinin resistance is prevalent and were largely absent in provinces where artemisinin resistance is not encountered. These molecular markers have already proved extraordinarily valuable in mapping the spread of artemisinin resistance across Myanmar, which is now perilously close to the border of India [9]. Indeed, they will likely revolutionize surveillance of artemisinin resistance and may provide more time and better information for devising strategies to mitigate the effects of resistance.

In the last few months two studies have investigated the physiological role of PfCRT and mechanism of PfKelch13-based artemisinin resistance, respectively. In the first of these studies, Moriyama and co-workers expressed recombinant PfCRT fused at the N- and C-terminal ends with a soluble *Escherichia coli* protein [10]. This was incorporated into liposomes. By varying the solution in which the liposomes were prepared as well as the solution in which they were dispersed together with the use of the potassium-selective ionophore valinomycin, the authors could explore the effects of pH and membrane potential on the transport of various substrates as well as antimalarials into the liposomes. Using radiolabeled tetraethyl ammonium ions (TEA), they demonstrated that substrate uptake into the liposomes required a pH gradient and membrane potential with lower pH and positive polarization on the outside of the liposome membrane. In the case of CQ-sensitive PfCRT^3D7^, a wide range of naturally occurring cationic species, including among others amino acids, peptides derived from hemoglobin degradation and glutathione, exerted a cis-inhibition of TEA transport into the liposomes. CQ, quinidine and the CQ-chemosensitizer verapamil had a similar effect. Furthermore, amino acids and CQ were shown to be taken up into the liposomes, demonstrating that PfCRT is capable of transporting these cationic substances across the membrane. Neutral and anionic amino acids and other biomolecules had no effect on TEA uptake and were not transported into the liposomes. This work suggests that the natural role of PfCRT is as a polyspecific organic cation transporter. Two surprises were that CQ was transported even by CQ-sensitive PfCRT and that much less cis-inhibition was exerted by this drug in the presence of the crucial K76T mutation in PfCRT that is required for resistance. Indeed, with PfCRT exhibiting the additional mutations present in CQ-resistant strains, namely PfCRT^7G8^ and PfCRT^Dd2^, CQ did not inhibit TEA transport. More detailed investigation indicated that CQ has lower affinity for mutant forms of PfCRT, but a higher maximal rate of transport (V_max_). This results in substantially faster CQ transport by CQ-resistant mutants of PfCRT. Finally, the results also hint at an additional role of CQ in inhibiting export of peptides derived from hemoglobin degradation from the DV.

In the second study, Haldar and co-workers used a fluorescent early endosome antigen 1 construct (SS-EEA1-mCherry) as a phosphatidylinositol-3-phosphate (PI3P) reporter [11]. In the absence of PI3P, wild-type SS-EEA1-mCherry is secreted from the endoplasmic reticulum to the parasitophorous vacuole of the parasite. This system had been used by the authors to screen for inhibitors of *Plasmodium falciparum* phosphatidylinositol-3-kinase (PfPI3K). They went on to show that dihydroartemisinin, the active metabolite of the artemisinins, inhibits PfPI3K in a reversible fashion in early ring stage parasites as visualized by the appearance of SS-EEA1-mCherry in the parasitophorous vacuole. The inactive analog, deoxyartemisinin caused no such effect so that PI3P formation occurred uninhibited and SS-EEA1-mCherry remained bound to PI3P in the endoplasmic reticulum. Artemisinins did not inhibit mammalian PI3K. Furthermore, homology modeling of the active site of PfPI3K indicated that dihydroartemisinin can make specific interactions with amino acids in this site. Parasites with mutant PfKelch13 associated with clinical resistance contained raised levels of PfPI3K. Wild-type PfKelch13 was shown to bind to PfPI3K and bring about ubiquitination of the PfPI3K, which results in protein degradation. The mutant forms of PfKelch13 were found not to bind PfPI3K, resulting in decreased ubiquitination and thus increased levels of the enzyme. Ring stage survival was found to correlate with raised PI3P levels.

While these studies are of undoubted scientific importance and will likely engender much new research and debate, it is also interesting to consider whether the implications of these findings are cause for optimism. Given that artemisinin resistance during the early ring stage is not related to any structural changes in the proposed PfPI3K target, but rather to increased concentration of this target arising from mutations in the PfKelch13 protein, prospects for avoiding resistance to this class of drug seem bleaker than might have been hoped. Only extraordinarily potent analogs or a substantial increase in dose would be likely to be able to overcome this form of resistance. Regarding drugs with CQ-like mechanisms of action, it would seem that these likely have a natural propensity to be transported by PfCRT and hence a predisposition for development of resistance. However, in this case it may at least in principle be possible to discover compounds that inhibit transport of cationic substrates without themselves being subject to such transport. If the findings with proteoliposomes are an accurate reflection of what happens in the DV, new thinking will thus be required in the design of any new drugs in this class.
